# Meta-analysis reveals associations between genetic variation in the 5′ and 3′ regions of Neuregulin-1 and schizophrenia

**DOI:** 10.1038/tp.2016.279

**Published:** 2017-01-17

**Authors:** M S Mostaid, S G Mancuso, C Liu, S Sundram, C Pantelis, I P Everall, C A Bousman

**Affiliations:** 1 Melbourne Neuropsychiatry Centre, Department of Psychiatry, The University of Melbourne, Carlton South, VIC, Australia; 2 Florey Institute of Neuroscience and Mental Health, The University of Melbourne, Parkville, VIC, Australia; 3 NorthWestern Mental Health, Melbourne Health, Parkville, VIC, Australia; 4 Centre for Neural Engineering (CfNE), Department of Electrical and Electronic Engineering, The University of Melbourne, Parkville, VIC, Australia; 5 Department of Psychiatry, School of Clinical Sciences, Monash University and Monash Health, Clayton, VIC, Australia; 6 Department of General Practice, The University of Melbourne, Parkville, VIC, Australia; 7 Swinburne University of Technology, Centre for Human Psychopharmacology, Hawthorn, VIC, Australia

## Abstract

Genetic, post-mortem and neuroimaging studies repeatedly implicate neuregulin-1 (*NRG1*) as a critical component in the pathophysiology of schizophrenia. Although a number of risk haplotypes along with several genetic polymorphisms in the 5′ and 3′ regions of *NRG1* have been linked with schizophrenia, results have been mixed. To reconcile these conflicting findings, we conducted a meta-analysis examining 22 polymorphisms and two haplotypes in *NRG1* among 16 720 cases, 20 449 controls and 2157 family trios. We found significant associations for three polymorphisms (rs62510682, rs35753505 and 478B14-848) at the 5′-end and two (rs2954041 and rs10503929) near the 3′-end of *NRG1.* Population stratification effects were found for the rs35753505 and 478B14-848(4) polymorphisms. There was evidence of heterogeneity for all significant markers and the findings were robust to publication bias. No significant haplotype associations were found. Our results suggest genetic variation at the 5′ and 3′ ends of *NRG1* are associated with schizophrenia and provide renewed justification for further investigation of *NRG1*’s role in the pathophysiology of schizophrenia.

## Introduction

Neuregulin-1 (NRG1) is a pleiotropic growth factor involved in circuitry generation, axon ensheathment, neuronal migration, synaptic plasticity, myelination and neurotransmission.^1, 2, 3, 4^ Thus, it is centrally involved in neurodevelopment and signalling in the mature central nervous system, where it exerts its actions through binding to its cognate receptor tyrosine kinases, ErbB3 and ErbB4, members of the epidermal growth factor system. The gene encoding *NRG1* is large, spanning ~1.2 Mb and contains >23 000 single-nucleotide polymorphisms (SNPs) among which ~40 have been associated with schizophrenia.^5^ Genome-wide association studies have generally, however, only provided modest support with the most recent study implicating rs986110 (*P*=1.5 × 10^−4^) with the disorder.^6^ This may in part be due to genome-wide association study to date focussing exclusively on SNP variation and consequently underestimating the importance of genes, such as *NRG1*, for which haplotype and microsatellite variation has been demonstrated. Thus, arguably a more thorough evaluation of *NRG1*’s association with schizophrenia requires examination of variation beyond SNPs.

Putative genetic/haplotypic variants in *NRG1* primarily sit within untranslated or intronic regions at the 5′ and 3′ ends of the gene. Yet, research to date has focused on the 5′-region of *NRG1*. This 5′-bias has been driven by the landmark study in 2002 conducted by Stefansson *et al.*,^7^ who identified a seven-marker schizophrenia-associated haplotype in the Icelandic population (HapICE) consisting of five SNPs and two microsatellites (478B14-848 and 420M9-1395) in the 5′-region of *NRG1*. As this milestone study, additional 5′-schizophrenia-associated haplotypes in the Irish (HapIRE)^8^ and Chinese (HapChina1-3)^9^ populations have been identified. However, the most recent meta-analysis conducted in 2008 (ref.^10^) only showed significant support for three (rs73235619, 478B14-848 and 420M9-1395) of the seven HapICE markers. Eight years have now passed since that meta-analysis and >20 case–control and family-based genetic association studies have been conducted. Moreover, the data required to conduct meta-analyses on genetic variation in the 3′-region of *NRG1* is now available. Thus, we have conducted an updated comprehensive meta-analysis of the association between *NRG1* genetic variation and schizophrenia, including single markers across the entire gene as well as haplotypes.

## Materials and methods

### Search strategy

The 2015 PRISMA-P (Preferred Reporting Items for Systematic review and Meta-Analysis Protocols) checklist^11^ was followed in reporting this meta-analysis. Studies were identified independently by two of the authors (MSM and CL) by searching three electronic databases: PubMed, PsychInfo and Medline (Ovid), using the search terms ‘neuregulin 1’, ‘neuregulin-1’, ‘neuregulin1’, ‘schizophrenia’ and ‘association’, and the abbreviation of the gene ‘NRG1’ and ‘NRG 1’ with no language restrictions. Bibliographies of all research articles were hand searched for additional references not indexed by MEDLINE. In cases where genotype data were not available in the published research articles or Supplementary Materials, we attempted to contact authors and request the required data. We also used the SZGene database (www.szgene.org) as a resource for collecting genotype data. All publications published from January 2002 through February 2016 were assessed for inclusion.

### Study selection and data extraction

For a study to be included in the meta-analysis, the following criteria were required: (a) a case–control or family-based genetic association studies investigating one or more SNPs and/or microsatellites of *NRG1*; (b) published in peer-reviewed journal containing original data; (c) included clinically diagnosed schizophrenia patients using an accepted classification system (for example, DSM and ICD); and (d) provided sufficient genotype or allelic data for calculation of an odds ratio (OR). Based on these criteria, 48 (40 case–control and 8 family-based) studies were included (Supplementary Figure S1; Table S1).

From each case–control and family study, the following data were extracted: (a) author(s) and publication year; (b) number of cases and controls or family sample size; (c) country of origin or ethnicity; (d) diagnostic criteria used; (e) SNP reference sequence number or marker identifier; (f) the publication identification number (for example, PubMed ID); (g) genotype counts and/or allele counts in cases and controls or family samples; and (h) haplotype frequencies in cases and controls (where available). Extracted data for all selected studies can be found in Supplementary File 2.

### Data synthesis and statistical analysis

Data from each case–control study were used to create 2 × 2 tables and data from each family study were used to create 1 × 2 tables. Classifications of the subjects were based on diagnostic category and type of allele they carried.

Data were analysed using *R* version 3.3.0 (R Foundation for Statistical Computing, Vienna, Austria). The meta^12^ and metafor^13^ packages were used to conduct the meta-analyses. The OR with 95% confidence intervals (CIs) was used as the effect size estimator. The method proposed by Kazeem and Farrall^14^ was used to calculate the effect size for transmission disequilibrium test studies, where the ORs were estimated from the number of transmissions versus non-transmissions of the designated high-risk allele to schizophrenia cases from heterozygous parents. For case–control studies, ORs were estimated by contrasting the ratio of counts of the high-risk versus low-risk alleles in schizophrenia cases versus non-clinical controls. For those polymorphisms in which the previous literature provided an indication of the risk-inducing allele, one-tailed *P*-values were reported. In the absence of prior data, two-tailed *P*-values were reported and were indicated accordingly in the text. All statistical tests (except for the *Q*-statistic) were considered statistically significant at *P*<0.05.

Because of the differences in study design and sample characteristics, considerable heterogeneity was expected between the studies. Therefore, the pooled OR was calculated using the random-effects models with the DerSimionian–Laird estimator,^15^ which is based on a normal distribution. The standard error estimates were adjusted using the Hartung–Knapp–Sidik–Jonkman^16, 17^ correction, which then calculates the corresponding 95% CI based on the *t*-distribution. The Hartung–Knapp–Sidik–Jonkman method generally outperforms the DerSimionian–Laird approach on type-I error rates when there is heterogeneity and the number of studies in the meta-analysis is small.^18, 19^

Outliers and influential studies were identified according to the recommendations of Viechtbauer and Cheung.^20^ Studies with observed effects that are well separated from the rest of the data are considered outliers. Such studies were identified using studentised deleted residuals, with absolute values >1.96 indicative of outliers. An influential study leads to considerable changes to the fitted model and a range of case deletion diagnostics adapted from linear regression can be used to identify these studies, including the DFFITS, DFBETAS and COVRATIO statistics (see Viechtbauer and Cheung^20^ for more information). Potential outliers and influential studies were omitted and the analyses were then re-run to determine their influence on the pooled effect size.

Heterogeneity in effect sizes across studies was tested using the *Q*-statistic (with *P*<0.10 indicating significant heterogeneity) and its magnitude was quantified using the *I*^2^ statistic, which is an index that describes the proportion of total variation in study effect size estimates that is due to heterogeneity and is independent of the number of studies included in the meta-analysis and the metric of effect sizes.^21^ As the *Q*-statistic has low power when the number of studies is small,^22^ 95% prediction intervals were calculated to quantify the extent of heterogeneity in the distribution of effect sizes.^23^ The prediction interval is an estimation of the range within which 95% of the true effect sizes are expected to fall.

Publication bias was assessed using funnel plots and the trim-and-fill procedure,^24^ which estimates the number of studies missing from the funnel plot and imputes these missing studies to make the funnel plot symmetrical, and then calculates an estimate of the effect size adjusted for publication bias.^25^ Following the recommendations of Sterne *et al.*,^26^ a test for funnel plot asymmetry was only conducted if the number of studies was 10 or greater. The regression test proposed by Harbord *et al.*^27^ was used to quantify the bias captured by the funnel plot and tested whether it was statistically significant. In addition, cumulative meta-analyses sorted by the sampling variance of the respective studies were conducted to examine the relationship between imprecise samples and effect sizes.^28^ This visualises the effect that small imprecise study samples have on the estimations of the pooled effect size.

The generalised linear mixed model method (that is, logistic regression) detailed in Bagos^29^ was used for the haplotype meta-analyses to avoid the inflation of the type-I error rate that is observed in the traditional approach of comparing a haplotype against the remaining ones.^29^

Moderator analyses for study design, diagnostic criteria and ancestry were conducted using mixed-effects meta-analyses. For this method, studies within potential moderator groups were pooled with the random-effects model, whereas tests for significant differences between the groups were conducted with the fixed-effects model. The Hartung–Knapp–Sidik–Jonkman adjustment was used if there were at least three studies in each group, otherwise the unadjusted DerSimionian–Laird method was used.

## Results

### Meta-analysis

**Figure 1:**
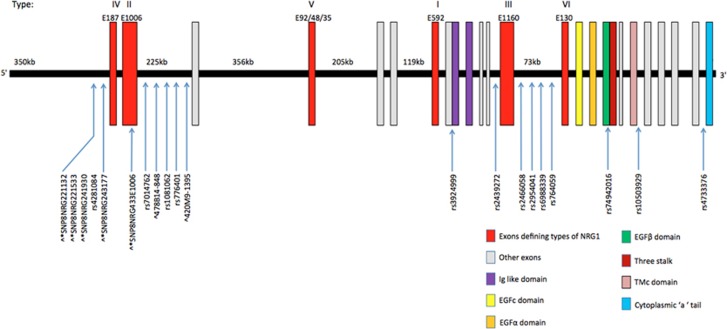
Location of NRG1 genetic variants included in the meta-analysis. *SNPs forming core ‘at-risk’ five-marker HapICE haplotype. ^Microsatellites in seven-marker HapICE haplotype. ^#^Markers shown to be significant in the current meta-analysis. HapICE, haplotype in the Icelandic population; SNP, single-nucleotide polymorphism.

A total of 22 single markers and two haplotypes that appeared in three or more studies were examined (Figure 1). Significant associations were found for three (rs62510682, 478B14-848(0) and rs2954041) of the 22 single markers but neither of the two haplotypes examined (Table 1; Supplementary Figures S2–S4).

### Heterogeneity, outlier and publication bias analysis

Across the three significant single markers, heterogeneity was low to moderate (*I*^2^=18.5–54.3%). The funnel plots are presented in Supplementary Figures S5–S7. The regression tests for funnel plot asymmetry were not statistically significant (Supplementary Table S2). Although the trim-and-fill method imputed two studies for rs62510682 and 478B14-848 (0), respectively, and three studies for rs2954041, the effect size adjusted for publication bias was comparable to the unadjusted effect size (Supplementary Table S2). The cumulative forest plots (Supplementary Figures S5–S10) also show that the point estimate stabilises with the inclusion of studies with smaller sampling variances. Taken together, this pattern of results suggests that the findings for the three significant single markers are likely robust to publication bias. Removal of potential outlier (that is, influential) studies in each of the meta-analyses produced small-to-moderate reductions in heterogeneity with minimal impact on the odd ratio (Supplementary Table S3). One exception was rs10503929, which after removal of an outlier study showed a significant association with schizophrenia (*k*=5, OR=1.14, 95% CI=1.10–1.18, *P⩽*0.001).

### Moderator analysis

Differential effects by study design, diagnostic criteria or ancestry were identified for two markers (Supplementary Table S3). The 4 allele of the 478B14-848 microsatellite had a ‘risk’ association among Asian studies (*k*=2, OR=1.18, 95% CI=1.01–1.38, *P*=0.021) and conversely a ‘protective’ association among European studies (*k*=3, OR=0.83, 95% CI=0.69–1.00, *P*=0.025; Supplementary Figure S11). Likewise, the rs35753505 (SNP8NRG221533) C-allele was associated with schizophrenia among Asian (*k*=12, OR=1.11, 95% CI=1.01–1.23, *P*=0.018) but not European (*k*=22, OR=1.01, 95% CI=0.94–1.09, *P*=0.376) studies (Supplementary Figure S12).

## Discussion

Three of the seven HapICE markers (rs62510682, rs35753505 and 478B14-848) at the 5′-end as well as two SNPs (rs2954041 and rs10503929) near the 3′-end of *NRG1* showed significant associations with schizophrenia. Our results concur with previous meta-analyses of *NRG1* that have reported associations for one or more of these markers (SZGene.org.),^10, 30, 31, 32, 33^ with the exception of the 3′ SNP rs2954041. To our knowledge, this is the first meta-analysis to identify an association between schizophrenia and rs2954041.

The rs2954041 SNP is located in the fifth intron of *NRG1*, ~18 kb from the type III (SMDF) promoter, the most brain abundant isoform of *NRG1.*^34^ To our knowledge, rs2954041 has not been assessed as expression quantitative trait loci for type III expression. However, given its proximal location to the type III promoter and preclinical evidence suggesting disruption of type III results in phenotypes commonly associated with schizophrenia (for example, enlarged ventricles and prepulse inhibition deficits),^35^ rs2954041 could have a functional role in the pathophysiology of schizophrenia. In addition, others have shown this SNP interacts with rs7424835 in *ERBB4*, the cognate receptor for *NRG1* (ref. 36) further highlighting a need to interrogate more comprehensively the 3′-end of *NRG1* in the context of schizophrenia. In fact, our results also showed the missense rs10503929 SNP, situated in exon 11 of the 3′-region, was associated with schizophrenia, although only after removal of an outlying family study.^37^ Importantly, our findings replicate those available in the SZGene database (www.szgene.org) and are based exclusively on studies within populations of European descent. This is notable because the rs10503929 ‘risk’ allele (T) is the major allele and is carried by all East Asians, 99% of Africans and 94% of South Asians relative to 81% of Europeans (http://browser.1000genomes.org/index.html). Thus, future studies in Asian and/or African populations may not be relevant or will require extremely large sample sizes.

Our findings from the 5′-end of *NRG1* that associate rs62510682, rs35753505 and 478B14-848 with schizophrenia have previously been identified in other meta-analyses. The rs35753505 is the most studied and the first *NRG1* marker to receive meta-analytic support for an association with schizophrenia.^30^ However, in three subsequent meta-analyses, this association was not detected.^10, 31, 32^ In the current meta-analysis, we have revived this association but only among Asians, which is contrary to the original meta-analytic association for rs35753505 that was found only among Caucasians.^30^ This finding is perhaps not surprising given evidence of population stratification at the *NRG1* locus.^10^ In fact, we also found that the 4 allele of the 478B14-848 microsatellite is a marker of ‘risk’ among Asians but ‘protection’ among Caucasians. This aligns with knowledge that the 0 allele in Asian populations is low^38, 39^ compared with the 4 allele, which is quite prevalent and forms in part the HapCHINA schizophrenia risk haplotype.^38, 39, 40^ However, no other markers we investigated were moderated by ancestry, including the three omnibus markers (rs62510682, 478B14-848(0) and rs2954041) associated with schizophrenia, albeit the number of non-Caucasian studies available for many of the markers hinders firm conclusions.

The rs62510682 (SNP8NRG241930) is the second most frequently studied *NRG1* marker but previous meta-analyses have been mixed. Li *et al.* showed in a meta-analysis of eight studies that carriers of the G allele had greater odds of a schizophrenia diagnosis, particularly among individuals of European descent; but in a subsequent meta-analysis of 14 studies by Gong *et al.*, this association was not upheld. Our meta-analysis of rs62510682 included 25 studies, a near doubling of the most recent meta-analysis, and reproduced the finding reported by Li *et al.* that suggests the G allele of rs62510682 is associated with schizophrenia. Our moderator analysis showed that this association did not differ by ancestry, although stratification analysis did suggest that this association might be stronger among individuals of European descent.

Although studied less frequently than other HapICE markers, the 0 ‘risk’ allele of the microsatellite 478B14-848 has been linked to schizophrenia in two previous meta-analyses,^10, 30^ although Li *et al.* combined carriers of the 0 and 4 alleles in their meta-analysis—an approach that has important implications with interpretation given our finding that the 4 allele can confer a ‘risk’ or ‘protective’ effect depending on ancestry. Nevertheless, our meta-analysis results uphold the meta-analytic association between the 0 allele and schizophrenia reported by Gong *et al.* and support further study of this potentially important microsatellite.

Our results, however, do not support an association between either the five- or seven-marker HapICE haplotypes and schizophrenia. To our knowledge, this is the first meta-analysis to examine the five- and seven-marker HapICE haplotypes. Although previous meta-analysis have showed positive associations for both five- and seven-marker haplotypes in schizophrenia,^10^ they pooled the results for non-identical five- and seven-marker haplotypes. Thus, their results do not reflect the overall association of the HapICE haplotype block in schizophrenia. Furthermore, most of the included studies were conducted in populations of European ancestry, which is not surprising given the frequency of the alleles that constitutes the HapICE risk haplotype is relatively low in Asian populations. In fact, most Asian studies do not look at the full HapICE haplotype but rather select SNPs and microsatellites forming the HapCHINA haplotype.

In conclusion, we have replicated and identified novel strong positive associations among polymorphisms situated at the 5′ and 3′ ends of *NRG1*. Although support for an association between the five- or seven-marker HapICE haplotypes and schizophrenia was not found, three of the markers within these haplotypes had robust associations. Our results highlight the importance of genetic variation at both the 5′ and 3′ ends of *NRG1* and provide justification for further investigation of NRG1’s role in the pathophysiology of schizophrenia.

## Figures and Tables

**Table 1:** Summary of single marker and haplotype meta-analyses

*NRG1 markers/haplotypes*	*Risk*	k	*Case/control (family trios)*	*Meta-analysis*				*Heterogeneity*			
				*OR*	*95% CI*	*90% CI*^a^	P	Q*/* τ^*2*^	P	I^*2*^	*95% PI*
*Single markers*											
rs73235619b,^c^	G	13	6145/6607 (262)	1.18	0.81, 1.71	0.87, 1.60	0.180	80.6	<0.001	85.1	0.47, 2.94
rs35753505b,^c^	C	35	12 708/14 302 (1601)	1.04	0.97, 1.11	0.98, 1.10	0.120	85.4	<0.001	60.2	0.79, 1.36
rs62510682b,^c^	G	25	10 791/11 986 (1248)	1.10	1.01, 1.20	1.02, 1.18	**0.018**	54.3	<0.001	55.8	0.84, 1.44
rs4281084	A	3	2217/2919	1.03	0.98, 1.08	1.00, 1.06	0.060	0.1	0.96	0.0	0.90, 1.18
rs6994992b,^c^	T	27	11 848/14 106 (1097)	1.00	0.96, 1.05	0.97, 1.04	0.440	36	0.09	27.8	0.88, 1.14
rs113317778b,^c^	G	10	4586/4935 (1003)	0.81	0.52, 1.25	0.57, 1.15	0.150	146.4	<0.001	93.9	0.17, 3.76
rs7014762	A	4	2128/2398 (634)	1.05	0.98, 1.12	1.00, 1.10	0.060	0.7	0.87	0.0	0.96, 1.15
478B14-848 (0)^c^	-	11	1071/1056 (111)	1.11	1.02, 1.20	**—**	**0.008**	12.3	0.27	18.5	0.97, 1.27
478B14-848 (4)	-	5	1015/894 (463)	0.98	0.74, 1.29	—	0.410	9.3	0.05	57.2	0.54, 1.78
rs1081062	C	4	2635/2946 (634)	0.99	0.83, 1.19	0.87, 1.14	0.460	3.6	0.31	16.1	0.71, 1.39
rs776401	C	3	3103/4817	0.97	0.63, 1.48	0.73, 1.29	0.390	10.3	0.006	80.6	0.12, 7.68
420M9-1395 (0)^c^	-	10	4777/4567 (111)	1.01	0.81, 1.25	—	0.460	36.5	<0.001	75.4	0.63, 1.63
420M9-1395 (-2)	-	7	1313/1130 (647)	1.05	0.96, 1.15	—	0.100	3.6	0.73	0.0	0.96, 1.15
rs3924999	A	16	6725/8551 (725+15bios)	1.02	0.90, 1.16	—	0.370	50.3	<0.001	70.2	0.72, 1.46
rs2439272	A	5	3003/4106 (111)	0.87	0.61, 1.23	0.67, 1.14	0.170	20.3	<0.001	80.3	0.42, 1.81
rs2466058	T	4	1863/1784 (111)	1.08	0.67, 1.74	0.76, 1.54	0.330	8.1	0.05	62.8	0.34, 3.39
rs2954041	T	7	3906/5527 (246)	1.21	0.97, 1.52	1.02, 1.45	**0.038**	10.6	0.10	43.3	0.76, 1.94
rs6988339	G	4	1113/2104 (111)	0.99	0.72, 1.37	0.78, 1.26	0.470	9.6	0.023	68.7	0.43, 2.27
rs764059	G	3	910/857 (111)	0.97	0.74, 1.28	0.81, 1.17	0.350	1.0	0.60	0.0	0.43, 2.18
rs74942016	T	3	1380/2222	1.11	0.77, 1.61	0.87, 1.43	0.170	0.3	0.87	0.0	0.38, 3.29
rs10503929	T	6	3399/4635 (151)	1.54	0.65, 3.65	0.78, 3.03	0.128	60.9	<0.001	91.8	0.36, 6.53
rs4733376	G	4	1843/2606 (111)	1.12	0.88, 1.42	0.94, 1.34	0.110	4.2	0.25	27.8	0.69, 1.81
											
*Haplotypes*											
Five-marker HapICE haplotype	GCGTG	5	2501/2283 (111)	1.16	0.91, 1.48	—	0.180	0.02^d^	0.07	53.1	0.67, 1.99
Seven-marker HapICE haplotype	GCGTG00	5	2501/2283 (111)	1.29	0.77, 2.13	—	0.250	0.12^d^	0.01	68.5	0.37, 4.39

Abbreviations: CI, confidence interval; HapICE, haplotype in the Icelandic population; OR, odds ratio; PI, prediction interval.

SNP8NRG221132=rs73235619, SNP8NRG221533=rs35753505, SNP8NRG241930=rs62510682, SNP8NRG243177=rs6994992, SNP8NRG433E1006=rs113317778. *P*<0.05 are bold faced.

a 90% CI for one-sided test.

b Markers forming five-marker HapICE haplotype.

c Markers forming seven-marker HapICE haplotype.

d Tau squared (*τ*^2^) values.
