# Supplementary material for: Meta-analysis reveals associations between genetic variation in the 5′ and 3′ regions of Neuregulin-1 and schizophrenia
Source: Transl Psychiatry. 2017 Jan 17;7(1):e1004–. doi: 10.1038/tp.2016.279 (PMC5545738; doi:10.1038/tp.2016.279)
Supplement: Supplementary Tables and Figures [file tp2016279x1.docx]

**SUPPLEMENTARY MATERIAL**

**Meta-analysis reveals associations between genetic variation in the 5’ and 3’ regions of neuregulin-1 and schizophrenia**

**Table of Contents**

**Supplementary Tables**

| **Table S1.** | Characteristics of included samples in the meta-analysis | **2** |
| --- | --- | --- |
| **Table S2.** | Publication bias analyses | **4** |
| **Table S3.** | Removal of potential outlier studies in each marker and subsequent meta-analysis | **5** |
| **Table S4.** | Moderator analyses of NRG1 markers | **6** |

**Supplementary Figures**

| **Figure S1** | Overview of the literature search and article screening procedure | **8** |
| --- | --- | --- |
| **Figure S2** | Forest plot of rs62510682 meta-analysis | **9** |
| **Figure S3** | Forest plot of microsatellite 478B14-848(0) meta-analysis | **10** |
| **Figure S4** | Forest plot of rs2954041 meta-analysis | **11** |
| **Figure S5** | Funnel plot of rs62510682 meta-analysis | **12** |
| **Figure S6** | Funnel plot of rs478B14-848(0) meta-analysis | **13** |
| **Figure S7** | Funnel plot of rs2954041 meta-analysis | **14** |
| **Figure S8** | Cumulative forest plot of rs62510682 meta-analysis | **15** |
| **Figure S9** | Cumulative forest plot of rs478B14-848(0) meta-analysis | **16** |
| **Figure S10** | Cumulative forest plot of rs2954041 meta-analysis | **17** |
| **Figure S11** | Forest plot of microsatellite 478B14-848(4) by ancestry | **18** |
| **Figure S12** | Forest plot of rs35753505 by ancestry | **19** |
| **References** |  | **20** |

**Supplementary Table S1:** Characteristics of included samples in the meta-analysis.

| **Author** | **Case/Control (family trios)** | **Ancestry** | **Method** | **Criteria** |
| --- | --- | --- | --- | --- |
| [Stefansson, Sigurdsson ^1^](#_ENREF_1) | 478/394 | Icelandic | Case-control | DSM-III-R |
| [Stefansson, Sarginson ^2^](#_ENREF_2) | 609/618 | Scottish | Case-control | DSM-III-R |
| [Yang, Si ^3^](#_ENREF_3) | 246 | Chinese | Family-based | ICD-10 |
| [Williams, Preece ^4^](#_ENREF_4) | 573/618 | Irish (UK) | Case-control | DSM-IV |
| [Iwata, Suzuki ^5^](#_ENREF_5) | 607/515 | Japanese | Case-control | DSM-IV |
| [Bakker, Hoogendoorn ^6^](#_ENREF_6) | 282/585 | Dutch | Case-control | DSM-IV |
| [Hong, Huo ^7^](#_ENREF_7) | 228/269 | Chinese | Case-control | DSM-IV |
| [Hong, Huo ^7^](#_ENREF_7) | 221 (15 bios) | Chinese | Family-based | DSM-IV |
| [Corvin, Morris ^8^](#_ENREF_8) | 243/222 | Irish | Case-control | DSM-IV |
| [Li, Stefansson ^9^](#_ENREF_9) | 298/336 | Chinese | Case-control | Mixed |
| [Li, Stefansson ^9^](#_ENREF_9) | 184 | Chinese | Family-based | Mixed |
| [Kampman, Anttila ^10^](#_ENREF_10) | 94/395 | Finnish | Case-control | DSM-IV |
| [Zhao, Shi ^11^](#_ENREF_11) | 369/299 | Chinese | Case-control | DSM-III-R |
| [Zhao, Shi ^11^](#_ENREF_11) | 352 | Chinese | Family-based | DSM-III-R |
| [Petryshen, Middleton ^12^](#_ENREF_12) | 321/242 | Portuguese | Case-control | DSM-IV |
| [Petryshen, Middleton ^12^](#_ENREF_12) | 111 | Portuguese | Family-based | DSM-IV |
| [Lachman, Pedrosa ^13^](#_ENREF_13) | 141/142 | African-American (USA) | Case-control | Mixed |
| [Lachman, Pedrosa ^13^](#_ENREF_13) | 177/164 | USA (Caucasian) | Case-control | Mixed |
| [Fukui, Muratake ^14^](#_ENREF_14) | 349/424 | Japanese | Case-control | DSM-IV |
| [Ingason, Soeby ^15^](#_ENREF_15) | 325/353 | Danish | Case-control | ICD-10 |
| [Kim, Lee ^16^](#_ENREF_16) | 242/242 | Korean | Case-control | DSM-IV |
| [Benzel, Bansal ^17^](#_ENREF_17) | 396/1342 | Caucasian (UK) | Case-control | OPCRIT |
| [Rosa, Gardner ^18^](#_ENREF_18) | 151 | Spanish | Family-based | DSM-IV |
| [Jungerius, Hoogendoorn ^19^](#_ENREF_19) | 310/880 | Netherlands | case-control | DSM-IV |
| [Georgieva, Dimitrova ^20^](#_ENREF_20) | 634 | Bulgarian | Family-based | DSM-IV |
| [Crowley, Keefe ^21^](#_ENREF_21) | 738/733 | USA (Mixed) | Case-control | DSM-IV |
| [Hanninen, Katila ^22^](#_ENREF_22) | 113/393 | Finnish | Case-control | DSM-IV |
| [Vilella, Costas ^23^](#_ENREF_23) | 589/615 | Spanish | Case-control | DSM-IV |
| [Ikeda, Takahashi ^24^](#_ENREF_24) | 1126/1022 | Japanese | Case-control | DSM-IV |
| [Shiota, Tochigi ^25^](#_ENREF_25) | 416/520 | Japanese | Case-control | DSM-IV |
| [Sanders, Duan ^26^](#_ENREF_26) | 1870/2002 | USA, Australia | Case-control | DSM-IV |
| [Bramon, Dempster ^27^](#_ENREF_27) | 64/35 | UK | Case-control | DSM-IV |
| [Hong, Wonodi ^28^](#_ENREF_28) | 244/186 | USA (Mixed) | Case-control | DSM-IV |
| [Alaerts, Ceulemans ^29^](#_ENREF_29) | 486/514 | Swedish | Case-control | DSM-IV |
| [Pedrosa, Nolan ^30^](#_ENREF_30) | 176/175 | USA (Caucasian) | Case-control | Mixed |
| [Zhang, Li ^31^](#_ENREF_31) | 258 | Chinese | Family-based | CCMD-3 |
| [Jonsson, Saetre ^32^](#_ENREF_32) | 837/1473 | Caucasian (Denmark, Norway, Sweden) | Case-control | Mixed |
| [So, Fong ^33^](#_ENREF_33) | 489/519 | Chinese | Case-control | DSM-IV |
| [Haraldsson, Ettinger ^34^](#_ENREF_34) | 113/106 | Icelandic | Case-control | RDC-SADS(L) |
| [Rethelyi, Bakker ^35^](#_ENREF_35) | 280/230 | Hungarian | Case-control | DSM-IV |
| [Squassina, Piccardi ^36^](#_ENREF_36) | 171/349 | Italian | Case-control | DSM-IV |
| (continued) |  |  |  |  |
| [Garcia-Barcelo, Miao ^37^](#_ENREF_37) | 270/270 | Chinese | Case-control | DSM-IV |
| [Naz, Riaz ^38^](#_ENREF_38) | 100/70 | Pakistani | Case-control | Unknown |
| [Moon, Rollins ^39^](#_ENREF_39) | 273/479 | Central Valley of Costa-Rica | Case-control | DSM-IV |
| [Papiol, Begemann ^40^](#_ENREF_40) | 1071/1056 | German (Caucasisan) | Case-control | DSM-IV-TR |
| [Mohamad Shariati, Behmanesh ^41^](#_ENREF_41) | 95/95 | Iranian | Case-control | DSM-IV |
| [Yang ^42^](#_ENREF_42) | 221/359 | Korean | Case-control | DSM-IV |
| [Kang, Yang ^43^](#_ENREF_43) | 287/120 | Chinese | Case-control | DSM-IV |
| [Crisafulli, Chiesa ^44^](#_ENREF_44) | 221/170 | Korean | Case-control | DSM-IV |
| [Weickert, Tiwari ^45^](#_ENREF_45) | 37/37 | Australian (Caucasian) | Case-control | DSM-IV |
| [Gutierrez-Fernandez, Palomino ^46^](#_ENREF_46) | 215/650 | Spanish | Case-control | DSM-IV |
| [Thirunavukkarasu, Vijayakumari ^47^](#_ENREF_47) | 38/37 | Indian | Case-control | DSM-IV |
| [Terzic, Kastelic ^48^](#_ENREF_48) | 138/94 | Slovenian | Case-control | DSM-IV |

**Supplementary Table S2:** Publication bias analyses.

| **Polymorphism** | **Risk** | **Meta-analysis** | | | | |  | **Regression Test** | |  | **Trim-and-Fill** | | | | |
| --- | --- | --- | --- | --- | --- | --- | --- | --- | --- | --- | --- | --- | --- | --- | --- |
|  |  | ***k*** | **OR** | **(95% CI)** | ***t*** | ***p*** |  | ***t*** | ***p*** |  | ***k_Imputed_*** | **OR** | **(95% CI)** | ***t*** | ***p*** |
| **rs62510682** | G | 27 | 1.00 | (0.96, 1.05) | 0.17 | 0.027 |  | 1.53 | 0.140 |  | 2 | 1.06 | (0.95, 1.18) | 1.13 | 0.027 |
| **478B14-848 (0)** | 0 | 10 | 1.08 | (1.01, 1.16) | 2.53 | 0.016 |  | 0.55 | 0.597 |  | 2 | 1.12 | (1.04, 1.21) | 3.21 | .008 |
| **rs2954041**^a^ | T | 6 | 1.13 | (1.00, 1.27) | 2.68 | 0.022 |  | - | - |  | 3 | 1.33 | (1.10, 1.62) | 3.31 | .009 |

^a^ Regression test not run due to k < 10.

**Supplementary Table S3:** Removal of potential outlier studies in each marker and subsequent meta-analysis.

| **Polymorphism** | **Risk** | **Meta-analysis** | | | | |  | **Heterogeneity** | | | **Study removed** |
| --- | --- | --- | --- | --- | --- | --- | --- | --- | --- | --- | --- |
|  |  | ***k*** | **OR** | **(95% CI)** | ***t*** | ***p*** |  | ***Q*** | ***p*** | ***I*^2^** |  |
| **rs73235619** | G | 12 | 1.02 | (0.91, 1.15) | 0.46 | 0.327 |  | 13.4 | 0.268 | 17.9 | [Rosa, Gardner ^18^](#_ENREF_18) |
| **rs35753505** | C | 34 | 1.02 | (0.97, 1.09) | 0.84 | 0.202 |  | 66.7 | <0.001 | 50.6 | [Zhang, Li ^31^](#_ENREF_31) |
| **rs62510682** | G | 27 | 1.00 | (0.96, 1.05) | 0.17 | 0.027 |  | 36.0 | 0.092 | 27.8 | [Rosa, Gardner ^18^](#_ENREF_18) |
| **rs6994992** | T | 26 | 0.99 | (0.95, 1.03) | 0.47 | 0.321 |  | 25.9 | 0.411 | 3.6 | [Stefansson, Sarginson ^2^](#_ENREF_2) |
| **rs113317778** | G | 9 | 0.98 | (0.80, 1.20) | 0.19 | 0.428 |  | 15.1 | 0.058 | 0.0 | [Zhang, Li ^31^](#_ENREF_31) |
| **478B14-848 (0)** | 0 | 10 | 1.08 | (1.01, 1.16) | 2.53 | 0.016 |  | 7.4 | 0.593 | 0.0 | [Iwata, Suzuki ^5^](#_ENREF_5) |
| **rs2439272** | A | 4 | 0.95 | (0.87, 1.03) | 2.17 | 0.059 |  | 1.4 | 0.701 | 0.0 | [Petryshen, Middleton ^12^](#_ENREF_12) |
| **rs4733376** | G | 3 | 1.05 | (0.96, 1.15) | 2.38 | 0.070 |  | 0.2 | 0.890 | 0.0 | [Benzel, Bansal ^17^](#_ENREF_17) |
| **rs2954041** | T | 6 | 1.13 | (1.00, 1.27) | 2.68 | 0.022 |  | 2.0 | 0.847 | 0.0 | [Yang, Si ^3^](#_ENREF_3) |
| **rs10503929** | T | 5 | 1.14 | (1.10, 1.18) | 10.11 | **<0.001** |  | 0.3 | 0.987 | 0.0 | Rosa, Gardner et al. (2007) |
| **rs7014762** | A | 3 | 1.03 | (0.95, 1.12) | 1.76 | 0.110 |  | 0.3 | 0.866 | 0.0 | [Georgieva, Dimitrova ^20^](#_ENREF_20) |

**Supplementary Table S4:** Moderator analyses of NRG1 markers.

| **Polymorphism** | **Risk** |  | **Meta-analysis** | | | | |  | **Heterogeneity** | | |  | **Moderator** | |
| --- | --- | --- | --- | --- | --- | --- | --- | --- | --- | --- | --- | --- | --- | --- |
|  |  |  | ***k*** | **OR** | **(95% CI)** | ***t*/*z*** | ***p*** |  | ***Q*** | ***p*** | ***I*^2^** |  | ***Q_M_*** | ***p*** |
| **rs73235619** | G |  |  |  |  |  |  |  |  |  |  |  |  |  |
| *Design* |  |  |  |  |  |  |  |  |  |  |  |  | 0.3 | 0.599 |
| Case-control |  |  | 11 | 1.03 | (0.95, 1.12) | 0.74 | 0.230 |  | 9.1 | 0.522 | 0.0 |  |  |  |
| TDT |  |  | 2 | 2.21 | (0.17, 28.40) | 0.61 | 0.272 |  | 52.6 | 0.000 | 98.1 |  |  |  |
| **rs35753505** | C |  |  |  |  |  |  |  |  |  |  |  |  |  |
| *Design* |  |  |  |  |  |  |  |  |  |  |  |  | 0.7 | 0.397 |
| Case-control |  |  | 30 | 1.02 | (0.97, 1.08) | 0.92 | 0.178 |  | 50.7 | 0.008 | 42.8 |  |  |  |
| TDT |  |  | 5 | 1.18 | (0.85, 1.64) | 1.00 | 0.158 |  | 33.8 | 0.000 | 88.2 |  |  |  |
| *Criteria* |  |  |  |  |  |  |  |  |  |  |  |  | 0.0 | 0.939 |
| DSM |  |  | 28 | 1.04 | (0.98, 1.10) | 1.37 | 0.086 |  | 49.2 | 0.006 | 45.1 |  |  |  |
| Other |  |  | 7 | 1.03 | (0.81, 1.31) | 0.23 | 0.410 |  | 36.2 | 0.000 | 83.4 |  |  |  |
| *Ancestry* |  |  |  |  |  |  |  |  |  |  |  |  | 2.4 | 0.124 |
| Asian |  |  | 12 | 1.11 | (1.01, 1.23) | 2.11 | 0.018 |  | 25.7 | 0.007 | 57.3 |  |  |  |
| European |  |  | 22 | 1.01 | (0.94, 1.09) | 0.32 | 0.376 |  | 49.4 | 0.000 | 57.5 |  |  |  |
| **rs62510682** | G |  |  |  |  |  |  |  |  |  |  |  |  |  |
| *Design* |  |  |  |  |  |  |  |  |  |  |  |  | 0.9 | 0.347 |
| Case-control |  |  | 21 | 1.07 | (1.01, 1.15) | 2.18 | 0.015 |  | 34.0 | 0.026 | 41.1 |  |  |  |
| TDT |  |  | 4 | 1.32 | (0.86, 2.04) | 1.27 | 0.101 |  | 19.5 | 0.000 | 84.6 |  |  |  |
| *Criteria* |  |  |  |  |  |  |  |  |  |  |  |  | 0.1 | 0.720 |
| DSM |  |  | 22 | 1.09 | (1.01, 1.18) | 2.29 | 0.011 |  | 46.8 | 0.001 | 55.1 |  |  |  |
| Other |  |  | 3 | 1.15 | (0.86, 1.55) | 0.96 | 0.168 |  | 7.5 | 0.023 | 73.5 |  |  |  |
| *Ancestry* |  |  |  |  |  |  |  |  |  |  |  |  | 0.3 | 0.578 |
| Asian |  |  | 8 | 1.04 | (0.89, 1.22) | 0.52 | 0.301 |  | 11.7 | 0.109 | 40.4 |  |  |  |
| European |  |  | 16 | 1.09 | (1.01, 1.18) | 2.31 | 0.011 |  | 34.1 | 0.003 | 56.0 |  |  |  |
| **rs6994992** | T |  |  |  |  |  |  |  |  |  |  |  |  |  |
| *Design* |  |  |  |  |  |  |  |  |  |  |  |  | 0.2 | 0.625 |
| Case-control |  |  | 24 | 1.00 | (0.95, 1.15) | 0.05 | 0.481 |  | 35.1 | 0.051 | 34.5 |  |  |  |
| TDT |  |  | 3 | 1.01 | (0.91, 1.17) | 0.54 | 0.294 |  | 0.6 | 0.724 | 0.0 |  |  |  |
| *Criteria* |  |  |  |  |  |  |  |  |  |  |  |  | 2.8 | 0.096 |
| DSM |  |  | 22 | 1.02 | (0.97, 1.07) | 0.86 | 0.195 |  | 27.0 | 0.170 | 22.3 |  |  |  |
| Other |  |  | 5 | 0.93 | (0.84, 1.03) | 1.44 | 0.076 |  | 5.0 | 0.285 | 20.4 |  |  |  |
| *Ancestry* |  |  |  |  |  |  |  |  |  |  |  |  | 0.1 | 0.712 |
| Asian |  |  | 9 | 1.00 | (0.94, 1.07) | 0.13 | 0.447 |  | 8.2 | 0.413 | 2.6 |  |  |  |
| European |  |  | 17 | 1.02 | (0.96, 1.08) | 0.71 | 0.240 |  | 23.4 | 0.103 | 31.7 |  |  |  |
| **rs113317778** | G |  |  |  |  |  |  |  |  |  |  |  |  |  |
| *Design* |  |  |  |  |  |  |  |  |  |  |  |  | 0.6 | 0.448 |
| Case-control |  |  | 7 | 0.98 | (0.81, 1.18) | 0.25 | 0.400 |  | 14.0 | 0.030 | 57.1 |  |  |  |
| TDT |  |  | 3 | 0.59 | (0.16, 2.17) | 0.80 | 0.211 |  | 49.3 | 0.000 | 95.9 |  |  |  |
| *Ancestry* |  |  |  |  |  |  |  |  |  |  |  |  | 1.1 | 0.302 |
| Asian |  |  | 2 | 0.41 | (0.08, 2.15) | -1.06 | 0.145 |  | 15.2 | 0.000 | 93.4 |  |  |  |
| European |  |  | 8 | 0.98 | (0.82, 1.17) | -0.22 | 0.414 |  | 15.1 | 0.035 | 53.5 |  |  |  |
|  |  |  |  |  |  |  |  |  |  |  |  |  |  |  |
| (continued) |  |  |  |  |  |  |  |  |  |  |  |  |  |  |
| **rs3924999** | A |  |  |  |  |  |  |  |  |  |  |  |  |  |
| *Design* |  |  |  |  |  |  |  |  |  |  |  |  | 0.1 | 0.753 |
| Case-control |  |  | 3 | 1.09 | (0.65, 1.83) | 0.33 | 0.370 |  | 23.0 | 0.000 | 91.3 |  |  |  |
| TDT |  |  | 13 | 1.00 | (0.92, 1.09) | 0.08 | 0.469 |  | 27.0 | 0.008 | 55.6 |  |  |  |
| *Criteria* |  |  |  |  |  |  |  |  |  |  |  |  | 0.0 | 0.894 |
| DSM |  |  | 11 | 1.02 | (0.94, 1.12) | 0.53 | 0.297 |  | 17.5 | 0.064 | 42.8 |  |  |  |
| Other |  |  | 5 | 1.04 | (0.79, 1.38) | 0.31 | 0.379 |  | 32.1 | 0.000 | 87.5 |  |  |  |
| *Ancestry* |  |  |  |  |  |  |  |  |  |  |  |  | 0.2 | 0.632 |
| Asian |  |  | 8 | 1.07 | (0.86, 1.33) | 0.59 | 0.279 |  | 39.9 | 0.000 | 82.4 |  |  |  |
| European |  |  | 7 | 1.01 | (0.93, 1.09) | 0.20 | 0.421 |  | 9.5 | 0.149 | 36.6 |  |  |  |
| **420M9-1395 (-2)** | (-2) |  |  |  |  |  |  |  |  |  |  |  |  |  |
| *Design* |  |  |  |  |  |  |  |  |  |  |  |  | 1.0 | 0.318 |
| Case-control |  |  | 4 | 1.02 | (0.89, 1.16) | 0.39 | 0.360 |  | 1.3 | 0.741 | 0.0 |  |  |  |
| TDT |  |  | 3 | 1.09 | (0.84, 1.43) | 1.47 | 0.140 |  | 1.7 | 0.427 | 0.0 |  |  |  |
| *Ancestry* |  |  |  |  |  |  |  |  |  |  |  |  | 0.6 | 0.457 |
| Asian |  |  | 4 | 1.07 | (0.95, 1.21) | 1.73 | 0.091 |  | 1.7 | 0.648 | 0.0 |  |  |  |
| European |  |  | 3 | 1.00 | (0.69, 1.44) | -0.02 | 0.491 |  | 1.5 | 0.462 | 0.0 |  |  |  |
| **478B14-848 (4)** | 4 |  |  |  |  |  |  |  |  |  |  |  |  |  |
| *Design* |  |  |  |  |  |  |  |  |  |  |  |  | 9.4 | 0.053 |
| Case-control |  |  | 3 | 0.98 | (0.78, 1.23) | 0.17 | 0.434 |  | 4.4 | 0.111 | 54.6 |  |  |  |
| TDT |  |  | 2 | 0.93 | (0.55, 1.56) | 0.29 | 0.387 |  | 4.9 | 0.027 | 79.6 |  |  |  |
| *Ancestry* |  |  |  |  |  |  |  |  |  |  |  |  | 8.0 | **0.005** |
| Asian |  |  | 2 | 1.18 | (1.01, 1.38) | 2.04 | 0.021 |  | 0.0 | 0.996 | 0.0 |  |  |  |
| European |  |  | 3 | 0.83 | (0.69, 1.00) | -1.97 | 0.025 |  | 1.4 | 0.503 | 0.0 |  |  |  |
| **rs2466058** | T |  |  |  |  |  |  |  |  |  |  |  |  |  |
| *Ancestry* |  |  |  |  |  |  |  |  |  |  |  |  | 1.5 | 0.221 |
| Asian |  |  | 2 | 0.95 | (0.70, 1.29) | -0.32 | 0.377 |  | 3.1 | 0.079 | 67.7 |  |  |  |
| European |  |  | 2 | 1.37 | (0.84, 2.24) | 1.24 | 0.107 |  | 1.7 | 0.190 | 41.9 |  |  |  |
| **rs1081062** | C |  |  |  |  |  |  |  |  |  |  |  |  |  |
| *Ancestry* |  |  |  |  |  |  |  |  |  |  |  |  | 1.2 | 0.266 |
| Asian |  |  | 2 | 1.12 | (0.86, 1.45) | 0.84 | 0.202 |  | 1.6 | 0.201 | 38.9 |  |  |  |
| European |  |  | 2 | 0.95 | (0.87, 1.05) | -0.94 | 0.173 |  | 0.1 | 0.744 | 0.0 |  |  |  |
| **rs2954041** | T |  |  |  |  |  |  |  |  |  |  |  |  |  |
| *Criteria* |  |  |  |  |  |  |  |  |  |  |  |  | 10.6 | 0.102 |
| DSM |  |  | 3 | 1.17 | (1.01, 1.36) | 2.03 | 0.022 |  | 0.3 | 0.840 | 0.0 |  |  |  |
| Other |  |  | 4 | 1.24 | (0.77, 1.99) | 0.87 | 0.192 |  | 8.8 | 0.032 | 66.0 |  |  |  |
| *Ancestry* |  |  |  |  |  |  |  |  |  |  |  |  | 1.8 | 0.174 |
| Asian |  |  | 4 | 1.33 | (1.02, 1.73) | 2.12 | 0.017 |  | 7.1 | 0.069 | 57.8 |  |  |  |
| European |  |  | 3 | 1.03 | (0.80, 1.33) | 0.23 | 0.408 |  | 0.9 | 0.634 | 0.0 |  |  |  |

Research articles identified through database searching

(n = 215)

Additional research articles identified by bibliographical search

(n = 12)

Abstract screened

(n = 227)

Studies included in meta-analysis

(n = 48)

Full-text articles assessed (n = 76)

Articles discarded for not meeting study criteria, (n = 28)

Articles discarded,

(n = 151)

**Supplementary Figure S1:** Overview of the literature search and article screening procedure


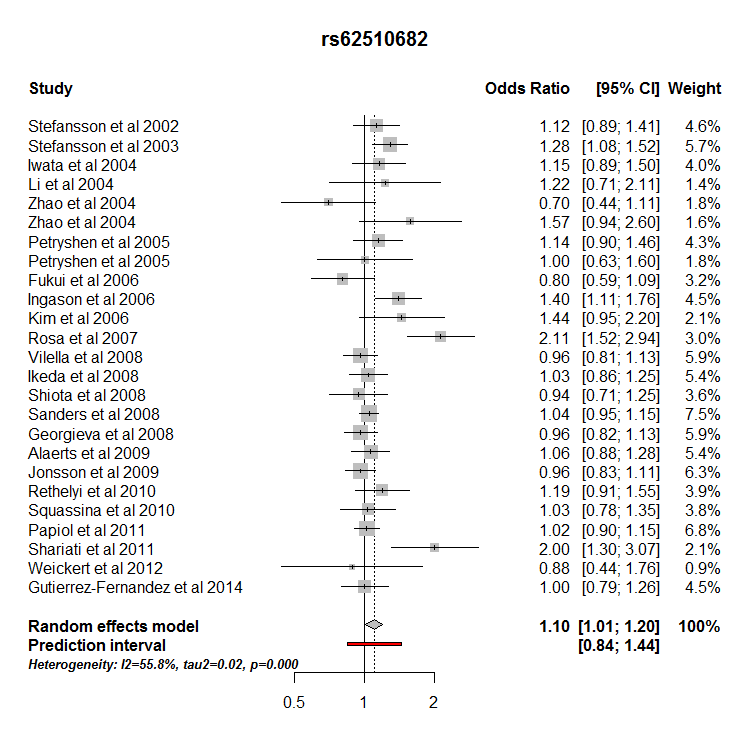


**Supplementary Figure S2:** Forest plot of rs62510682 meta-analysis.


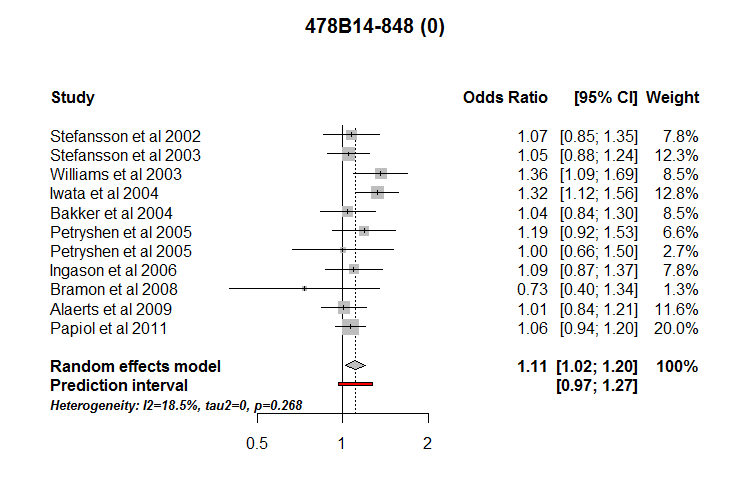


**Supplementary Figure S3:** Forest plot of microsatellite 478B14-848(0) meta-analysis.


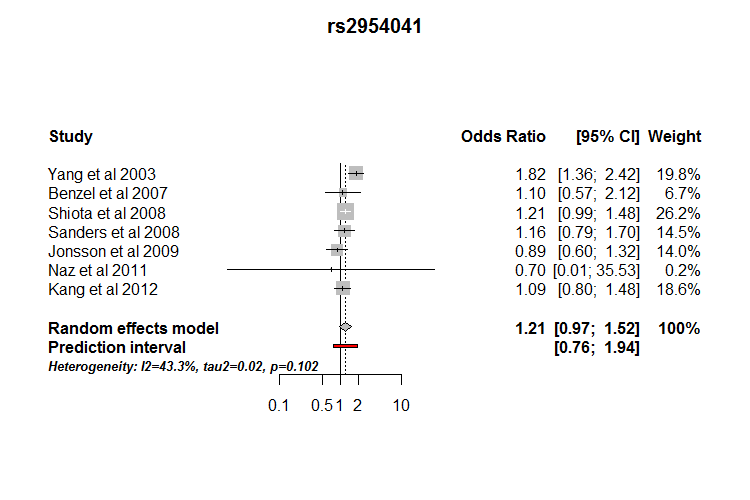


**Supplementary Figure S4:** Forest plot of rs2954041 meta-analysis


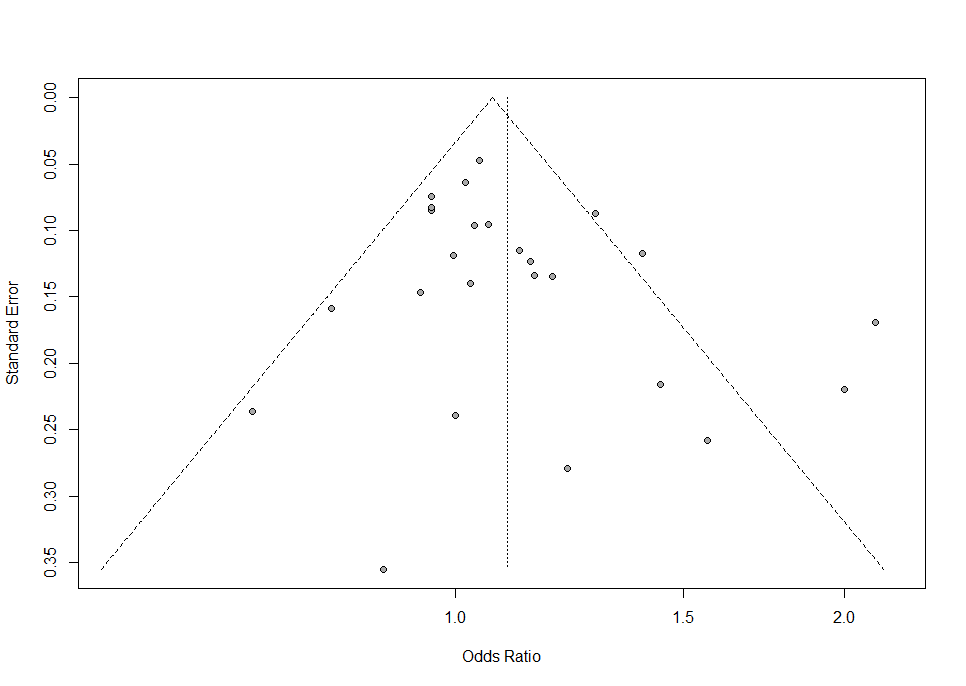


**Supplementary Figure S5:** Funnel plot of rs62510682 meta-analysis.


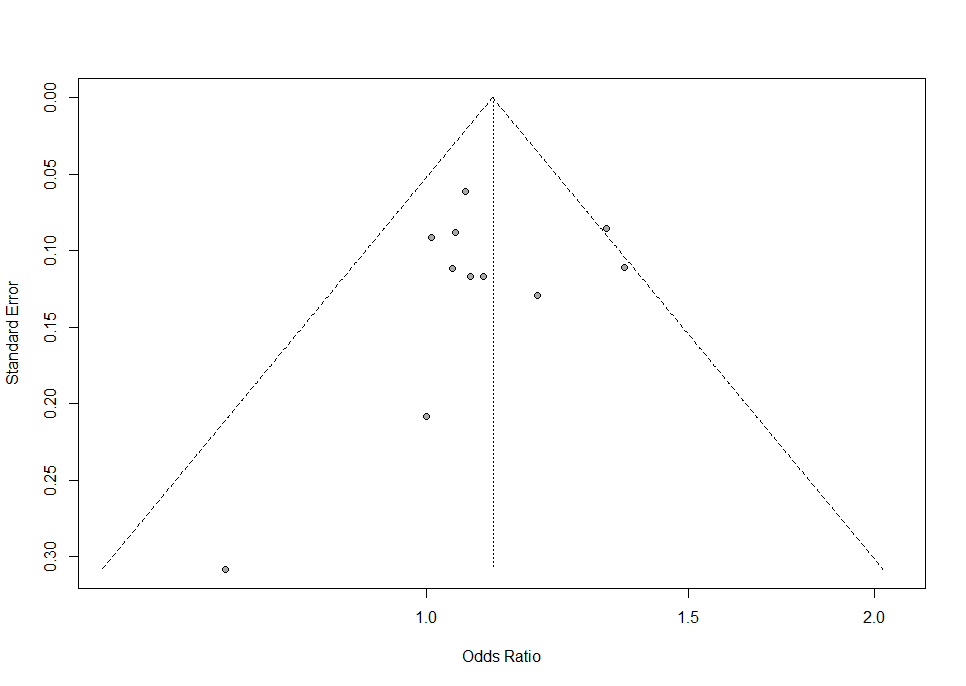


**Supplementary Figure S6:** Funnel plot of rs478B14-848 (0) meta-analysis.


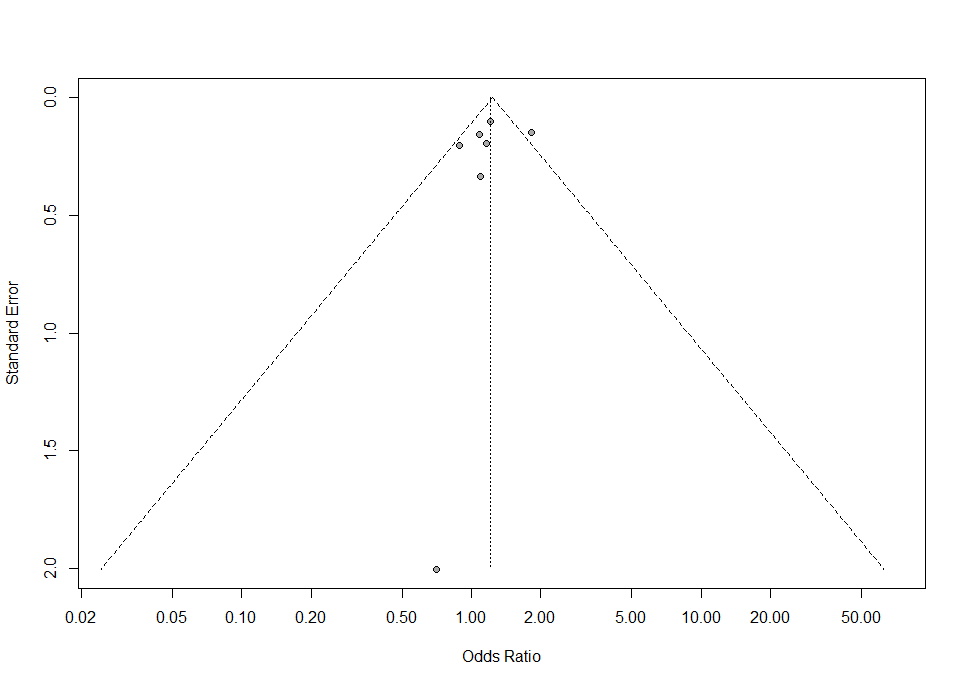


**Supplementary Figure S7:** Funnel plot of rs2954041 meta-analysis.


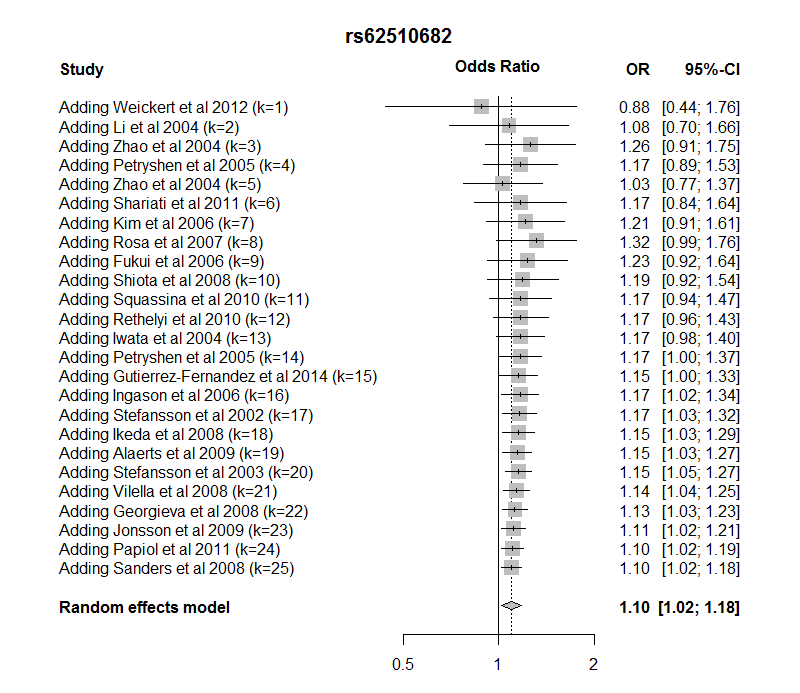


**Supplementary Figure S8:** Cumulative forest plot of rs62510682 meta-analysis.


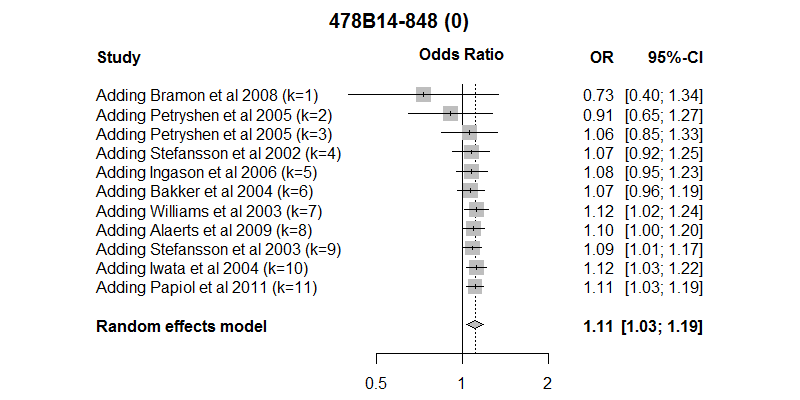


**Supplementary Figure S9:** Cumulative forest plot of rs478B14-848 (0) meta-analysis.


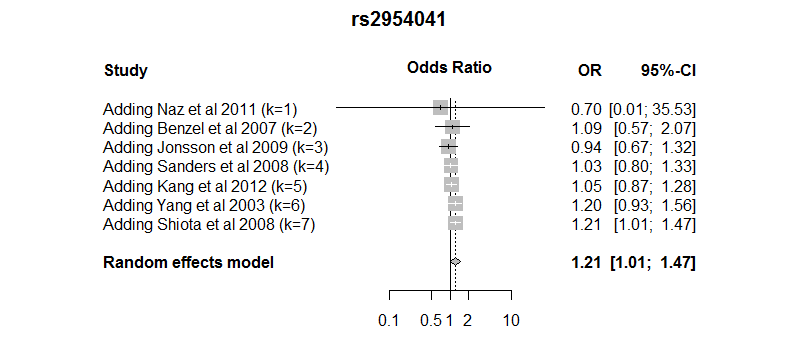


**Supplementary Figure S10:** Cumulative forest plot of rs2954041 meta-analysis.


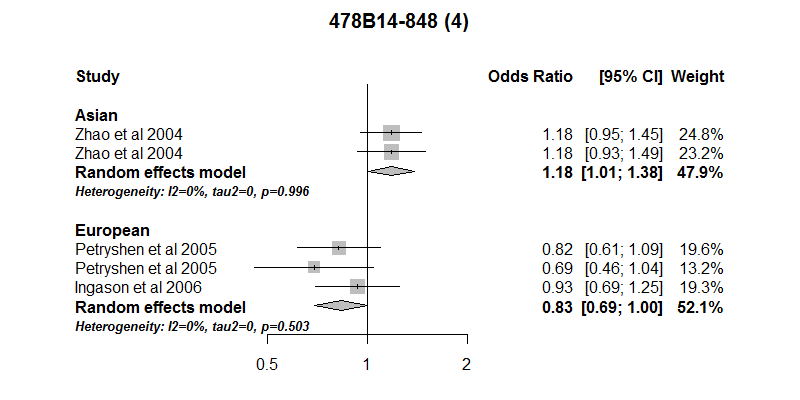


**Supplementary Figure S11:** Forest plot of microsatellite 478B14-848 (4) by ancestry.


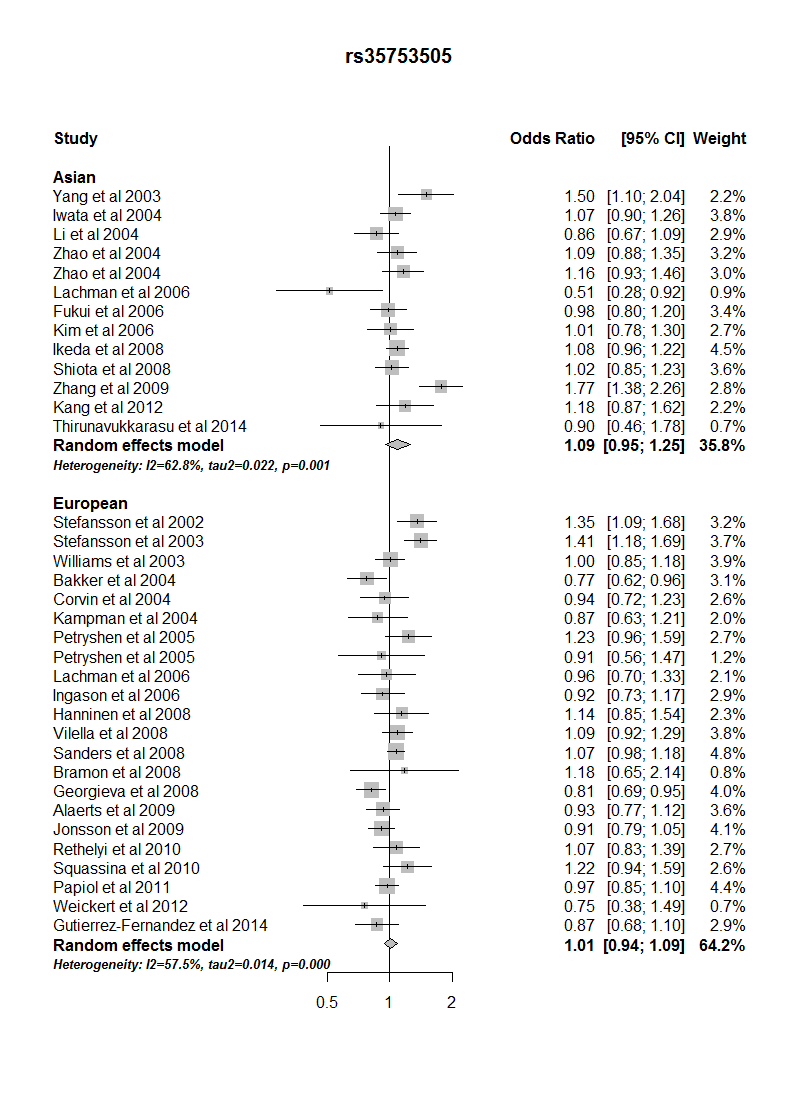


**Supplementary Figure S12:** Forest plot of rs35753505 by ancestry.

**References**

1. Stefansson H, Sigurdsson E, Steinthorsdottir V, Bjornsdottir S, Sigmundsson T, Ghosh S *et al.* Neuregulin 1 and susceptibility to schizophrenia. *Am J Hum Genet* 2002; **71**(4)**:** 877-892.

2. Stefansson H, Sarginson J, Kong A, Yates P, Steinthorsdottir V, Gudfinnsson E *et al.* Association of neuregulin 1 with schizophrenia confirmed in a Scottish population. *Am J Hum Genet* 2003; **72**(1)**:** 83-87.

3. Yang JZ, Si TM, Ruan Y, Ling YS, Han YH, Wang XL *et al.* Association study of neuregulin 1 gene with schizophrenia. *Mol Psychiatry* 2003; **8**(7)**:** 706-709.

4. Williams NM, Preece A, Spurlock G, Norton N, Williams HJ, Zammit S *et al.* Support for genetic variation in neuregulin 1 and susceptibility to schizophrenia. *Mol Psychiatry* 2003; **8**(5)**:** 485-487.

5. Iwata N, Suzuki T, Ikeda M, Kitajima T, Yamanouchi Y, Inada T *et al.* No association with the neuregulin 1 haplotype to Japanese schizophrenia. *Mol Psychiatry* 2004; **9**(2)**:** 126-127.

6. Bakker SC, Hoogendoorn ML, Selten JP, Verduijn W, Pearson PL, Sinke RJ *et al.* Neuregulin 1: genetic support for schizophrenia subtypes. *Mol Psychiatry* 2004; **9**(12)**:** 1061-1063.

7. Hong CJ, Huo SJ, Liao DL, Lee K, Wu JY, Tsai SJ. Case-control and family-based association studies between the neuregulin 1 (Arg38Gln) polymorphism and schizophrenia. *Neurosci Lett* 2004; **366**(2)**:** 158-161.

8. Corvin AP, Morris DW, McGhee K, Schwaiger S, Scully P, Quinn J *et al.* Confirmation and refinement of an 'at-risk' haplotype for schizophrenia suggests the EST cluster, Hs.97362, as a potential susceptibility gene at the Neuregulin-1 locus. *Mol Psychiatry* 2004; **9**(2)**:** 208-213.

9. Li T, Stefansson H, Gudfinnsson E, Cai G, Liu X, Murray RM *et al.* Identification of a novel neuregulin 1 at-risk haplotype in Han schizophrenia Chinese patients, but no association with the Icelandic/Scottish risk haplotype. *Mol Psychiatry* 2004; **9**(7)**:** 698-704.

10. Kampman O, Anttila S, Illi A, Saarela M, Rontu R, Mattila KM *et al.* Neuregulin genotype and medication response in Finnish patients with schizophrenia. *Neuroreport* 2004; **15**(16)**:** 2517-2520.

11. Zhao X, Shi Y, Tang J, Tang R, Yu L, Gu N *et al.* A case control and family based association study of the neuregulin1 gene and schizophrenia. *J Med Genet* 2004; **41**(1)**:** 31-34.

12. Petryshen TL, Middleton FA, Kirby A, Aldinger KA, Purcell S, Tahl AR *et al.* Support for involvement of neuregulin 1 in schizophrenia pathophysiology. *Mol Psychiatry* 2005; **10**(4)**:** 366-374, 328.

13. Lachman HM, Pedrosa E, Nolan KA, Glass M, Ye K, Saito T. Analysis of polymorphisms in AT-rich domains of neuregulin 1 gene in schizophrenia. *Am J Med Genet B Neuropsychiatr Genet* 2006; **141B**(1)**:** 102-109.

14. Fukui N, Muratake T, Kaneko N, Amagane H, Someya T. Supportive evidence for neuregulin 1 as a susceptibility gene for schizophrenia in a Japanese population. *Neurosci Lett* 2006; **396**(2)**:** 117-120.

15. Ingason A, Soeby K, Timm S, Wang AG, Jakobsen KD, Fink-Jensen A *et al.* No significant association of the 5' end of neuregulin 1 and schizophrenia in a large Danish sample. *Schizophr Res* 2006; **83**(1)**:** 1-5.

16. Kim JW, Lee YS, Cho EY, Jang YL, Park DY, Choi KS *et al.* Linkage and association of schizophrenia with genetic variations in the locus of neuregulin 1 in Korean population. *Am J Med Genet B Neuropsychiatr Genet* 2006; **141B**(3)**:** 281-286.

17. Benzel I, Bansal A, Browning BL, Galwey NW, Maycox PR, McGinnis R *et al.* Interactions among genes in the ErbB-Neuregulin signalling network are associated with increased susceptibility to schizophrenia. *Behav Brain Funct* 2007; **3:** 31.

18. Rosa A, Gardner M, Cuesta MJ, Peralta V, Fatjo-Vilas M, Miret S *et al.* Family-based association study of neuregulin-1 gene and psychosis in a Spanish sample. *Am J Med Genet B Neuropsychiatr Genet* 2007; **144B**(7)**:** 954-957.

19. Jungerius BJ, Hoogendoorn ML, Bakker SC, Van't Slot R, Bardoel AF, Ophoff RA *et al.* An association screen of myelin-related genes implicates the chromosome 22q11 PIK4CA gene in schizophrenia. *Mol Psychiatry* 2008; **13**(11)**:** 1060-1068.

20. Georgieva L, Dimitrova A, Ivanov D, Nikolov I, Williams NM, Grozeva D *et al.* Support for neuregulin 1 as a susceptibility gene for bipolar disorder and schizophrenia. *Biol Psychiatry* 2008; **64**(5)**:** 419-427.

21. Crowley JJ, Keefe RS, Perkins DO, Stroup TS, Lieberman JA, Sullivan PF. The neuregulin 1 promoter polymorphism rs6994992 is not associated with chronic schizophrenia or neurocognition. *Am J Med Genet B Neuropsychiatr Genet* 2008; **147B**(7)**:** 1298-1300.

22. Hanninen K, Katila H, Saarela M, Rontu R, Mattila KM, Fan M *et al.* Interleukin-1 beta gene polymorphism and its interactions with neuregulin-1 gene polymorphism are associated with schizophrenia. *Eur Arch Psychiatry Clin Neurosci* 2008; **258**(1)**:** 10-15.

23. Vilella E, Costas J, Sanjuan J, Guitart M, De Diego Y, Carracedo A *et al.* Association of schizophrenia with DTNBP1 but not with DAO, DAOA, NRG1 and RGS4 nor their genetic interaction. *J Psychiatr Res* 2008; **42**(4)**:** 278-288.

24. Ikeda M, Takahashi N, Saito S, Aleksic B, Watanabe Y, Nunokawa A *et al.* Failure to replicate the association between NRG1 and schizophrenia using Japanese large sample. *Schizophr Res* 2008; **101**(1-3)**:** 1-8.

25. Shiota S, Tochigi M, Shimada H, Ohashi J, Kasai K, Kato N *et al.* Association and interaction analyses of NRG1 and ERBB4 genes with schizophrenia in a Japanese population. *J Hum Genet* 2008; **53**(10)**:** 929-935.

26. Sanders AR, Duan J, Levinson DF, Shi J, He D, Hou C *et al.* No significant association of 14 candidate genes with schizophrenia in a large European ancestry sample: implications for psychiatric genetics. *Am J Psychiatry* 2008; **165**(4)**:** 497-506.

27. Bramon E, Dempster E, Frangou S, Shaikh M, Walshe M, Filbey FM *et al.* Neuregulin-1 and the P300 waveform--a preliminary association study using a psychosis endophenotype. *Schizophr Res* 2008; **103**(1-3)**:** 178-185.

28. Hong LE, Wonodi I, Stine OC, Mitchell BD, Thaker GK. Evidence of missense mutations on the neuregulin 1 gene affecting function of prepulse inhibition. *Biol Psychiatry* 2008; **63**(1)**:** 17-23.

29. Alaerts M, Ceulemans S, Forero D, Moens LN, De Zutter S, Heyrman L *et al.* Support for NRG1 as a susceptibility factor for schizophrenia in a northern Swedish isolated population. *Arch Gen Psychiatry* 2009; **66**(8)**:** 828-837.

30. Pedrosa E, Nolan KA, Stefanescu R, Herskovits P, Novak T, Zukov I *et al.* Analysis of a promoter polymorphism in the SMDF neuregulin 1 isoform in Schizophrenia. *Neuropsychobiology* 2009; **59**(4)**:** 205-212.

31. Zhang HX, Li WQ, Zhang Y, Zhao JP, Lv LX, Yang G. [Association analysis of neuregulin 1 gene polymorphism with schizophrenia in Chinese Han population]. *Zhonghua Yi Xue Yi Chuan Xue Za Zhi* 2009; **26**(1)**:** 16-20.

32. Jonsson EG, Saetre P, Vares M, Andreou D, Larsson K, Timm S *et al.* DTNBP1, NRG1, DAOA, DAO and GRM3 polymorphisms and schizophrenia: an association study. *Neuropsychobiology* 2009; **59**(3)**:** 142-150.

33. So HC, Fong PY, Chen RY, Hui TC, Ng MY, Cherny SS *et al.* Identification of neuroglycan C and interacting partners as potential susceptibility genes for schizophrenia in a Southern Chinese population. *Am J Med Genet B Neuropsychiatr Genet* 2010; **153B**(1)**:** 103-113.

34. Haraldsson HM, Ettinger U, Magnusdottir BB, Ingason A, Hutton SB, Sigmundsson T *et al.* Neuregulin-1 genotypes and eye movements in schizophrenia. *Eur Arch Psychiatry Clin Neurosci* 2010; **260**(1)**:** 77-85.

35. Rethelyi JM, Bakker SC, Polgar P, Czobor P, Strengman E, Pasztor PI *et al.* Association study of NRG1, DTNBP1, RGS4, G72/G30, and PIP5K2A with schizophrenia and symptom severity in a Hungarian sample. *Am J Med Genet B Neuropsychiatr Genet* 2010; **153B**(3)**:** 792-801.

36. Squassina A, Piccardi P, Del Zompo M, Rossi A, Vita A, Pini S *et al.* NRG1 and BDNF genes in schizophrenia: an association study in an Italian case-control sample. *Psychiatry Res* 2010; **176**(1)**:** 82-84.

37. Garcia-Barcelo MM, Miao X, Tang CS, So HC, Tang W, Leon TY *et al.* No NRG1 V266L in Chinese patients with schizophrenia. *Psychiatr Genet* 2011; **21**(1)**:** 47-49.

38. Naz M, Riaz M, Saleem M. Potential role of Neuregulin 1 and TNF-alpha (-308) polymorphism in schizophrenia patients visiting hospitals in Lahore, Pakistan. *Mol Biol Rep* 2011; **38**(7)**:** 4709-4714.

39. Moon E, Rollins B, Mesen A, Sequeira A, Myers RM, Akil H *et al.* Lack of association to a NRG1 missense polymorphism in schizophrenia or bipolar disorder in a Costa Rican population. *Schizophr Res* 2011; **131**(1-3)**:** 52-57.

40. Papiol S, Begemann M, Rosenberger A, Friedrichs H, Ribbe K, Grube S *et al.* A phenotype-based genetic association study reveals the contribution of neuregulin1 gene variants to age of onset and positive symptom severity in schizophrenia. *Am J Med Genet B Neuropsychiatr Genet* 2011; **156B**(3)**:** 340-345.

41. Mohamad Shariati SA, Behmanesh M, Galehdari H. A Study of the Association between SNP8NRG241930 in the 5' End of Neuroglin 1 Gene with Schizophrenia in a Group of Iranian Patients. *Cell journal* 2011; **13**(2)**:** 91-96.

42. Yang SA. Association between a Missense Polymorphism (rs3924999, Arg253Gln) of Neuregulin 1 and Schizophrenia in Korean Population. *Exp Neurobiol* 2012; **21**(4)**:** 158-163.

43. Kang C, Yang X, Xu X, Liu H, Su P, Yang J. Association study of neuregulin 1 gene polymorphisms with auditory P300 in schizophrenia. *Am J Med Genet B Neuropsychiatr Genet* 2012; **159B**(4)**:** 422-428.

44. Crisafulli C, Chiesa A, Han C, Lee SJ, Park MH, Balzarro B *et al.* Case-control association study for 10 genes in patients with schizophrenia: influence of 5HTR1A variation rs10042486 on schizophrenia and response to antipsychotics. *Eur Arch Psychiatry Clin Neurosci* 2012; **262**(3)**:** 199-205.

45. Weickert CS, Tiwari Y, Schofield PR, Mowry BJ, Fullerton JM. Schizophrenia-associated HapICE haplotype is associated with increased NRG1 type III expression and high nucleotide diversity. *Translational psychiatry* 2012; **2:** e104.

46. Gutierrez-Fernandez A, Palomino A, Gonzalez-Pinto A, Ugarte A, Hernanz M, Mendibil B *et al.* Novel association of Neuregulin 1 gene with bipolar disorder but not with schizophrenia. *Schizophr Res* 2014.

47. Thirunavukkarasu P, Vijayakumari AA, John JP, Halahalli HN, Paul P, Sen S *et al.* An exploratory association study of the influence of dysbindin and neuregulin polymorphisms on brain morphometry in patients with schizophrenia and healthy subjects from South India. *Asian J Psychiatr* 2014; **10:** 62-68.

48. Terzic T, Kastelic M, Dolzan V, Plesnicar BK. Genetic variability testing of neurodevelopmental genes in schizophrenic patients. *J Mol Neurosci* 2015; **56**(1)**:** 205-211.
